# A Bibliometric-Based Analytical Framework for the Study of Smart City Lifeforms in China

**DOI:** 10.3390/ijerph192214762

**Published:** 2022-11-10

**Authors:** Yanmin Xu, Wengang Li, Jianjiang Tai, Chunjiong Zhang

**Affiliations:** 1 China Special Economic Zone Research Center, Shenzhen University, Shenzhen 518061, China; 2 School of Marxism, Shenzhen Institute of Administration, Shenzhen 518034, China; 3 School of Humanities, Shanghai University of Finance and Economics, Shanghai 200433, China; 4 Department of Computer Science, Tongji University, Shanghai 201804, China

**Keywords:** smart city, co-word analysis, digitization, humanistic city, urban lifeform

## Abstract

Smart cities are the future development direction of cities and are a comprehensive expression of the development of the organic life body of cities. The organic life form of a smart city relates to viewing the city as an organic life self-organizing system based on the wholeness and systemic nature of the smart city life form itself, to construct a holistic spatial linkage of the functions and mechanisms of the city life system, and to enhance the overall vitality of the space. This study is based on the literature of “smart city” research in the China National Knowledge Infrastructure (CNKI) database, and the current situation and related themes of smart city research in China are discussed through co-word analysis and cluster analysis using software such as SPSS and VOSviewer, among which there are four themes in co-word cluster analysis, namely, intelligent technology supporting smart city research; research on the integration of the social system of a smart city; research on the top-level strategic design and planning and construction of a smart city; and research on the development, evaluation, and concrete practice of smart city construction. Four conclusions are drawn from the development of smart city research in China: Firstly, smart city research has attracted the attention of multiple disciplines, and the research themes are scattered and integrated across disciplinary systems. Secondly, smart city construction, development rules, and characteristics need to be further explored, and the problems, future trends, and policy support for the modernization of China’s cities and towns have been focused on engineering and technology, with a lack of practical research in non-technical areas such as humanities and ethics. Thirdly, the philosophical humanism and ecological ethics of smart cities need to be systematized, and their construction and development needs to be humanistic, systematic, and comprehensive, thus contributing to the sustainability, livability, ecology, and wisdom of future urban development. Fourthly, the development of the smart city system is supported by theories related to global cities and innovative cities, and the world city, a product of globalization, is undergoing a transformation into a digital and intelligent organic urban life form.

## 1. Introduction

Since International Business Machines Corporation (IBM) put forward the concept of a “smart earth” in 2008, with the spatial application of information and intelligent technology, the term smart city has gradually become synonymous with the future development of the city. In 2012, China promulgated the Notice on Carrying Out the National Smart City Pilot Work, which marked the beginning of the specific practical work of China’s smart city construction. China’s smart city construction has experienced more than ten years of development. At present, the relevant themes need to be grasped as a whole, so as to summarize the development law, enrich the theoretical framework, and provide corresponding guidance for specific practical problems. Smart city construction promotes the optimization of urban ecosystems with intelligent perception and interconnection integration from five aspects: concept strategy, social development, economic development, spatial planning, and infrastructure, so as to provide corresponding methods and strategies to solve the problems faced by urban development [1]. Learning from the sustainable, big data, and other diversified smart city models shaped in the construction of global smart cities could promote the coordination of smart city governance, personalized urban services for people, and the comprehensive aggregation of technology applications, releasing the dividends of corresponding policies and systems and then grasping the new trend of smart city construction [2].

It is clear that smart cities are of significant value to the current problems and challenges encountered in urban development, and a large number of research results have emerged. The technological revolution has led to changes in traditional production and lifestyles, profoundly altering the way society is organized and the way urban space operates, shaping a whole new pattern of human urban social systems. Smart cities driven by digital and intelligent technologies have emerged, and the development of smart city construction relies on the interaction process between modern intelligent technologies and urban development needs. However, the specific objectives and action plans of smart city construction are applied piecemeal in the field of urban construction and rely excessively on the technical sphere, forcing people to experience unsatisfactory results and other real problems that need to be addressed accordingly. Based on this, this paper analyzes the state of smart city research in China, further explores the evolution of smart city research paradigms, tracks research frontiers and research hotspots, and provides reference and guidance for future smart city practice and theoretical research.

## 2. Literature Review

From the specific practice of smart city development, it has become mainstream to build and develop smart cities around technology support. Governments around the world are focusing on smart city projects to achieve diverse goals such as sustainability, citizen engagement, and improved services [3]. The development of smart city applications involves the improvement of the quality of life of residents in society and the use of cloud computing for the sensing infrastructure data calculation to achieve the application of allowing the process of activity to unfold through the encryption of computing methods to achieve data privacy protection and to help the development of smart city applications [4].

From the perspective of the theoretical development of a smart city, the theoretical connotation of a smart city and its technical support framework have undergone changes in specific practice. Smart City Version 1.0 is a smart urban solution promoted by technology companies; Smart City Version 2.0 is a local government solution leading the improvement of residents’ lives and sustainable lifestyles; Smart City Version 3.0 is a community collaboration model, which includes the best strategic solutions for citizens, policy makers, enterprises, and other subjects [5]. The development of smart city theory cannot be separated from the logic of specific technological developments, where technologies such as the Internet of Things, big data, and artificial intelligence have become the underlying framework supporting and influencing the connotative development of smart city theory, here including the theoretical categories of sustainability, innovation, and governance. Geo-Twitter provides information on the perceived experience of smart cities in Australia, where Sydney, Melbourne, and Brisbane are the leading smart cities, by providing appropriate decision-making information for smart city development at a community level [6]. The conceptual goals of a smart city are to provide quality services to city residents and to make the best use of public resources to improve people’s quality of life. Blockchain technology is applied in the smart city service system, using its advantages of decentralization, anonymity, and security to analyze smart city application areas such as smart transportation, smart healthcare, and supply chain management [7]. The Neoliberalism of Community Citizen Participation in Smart Cities links different forms of neoliberal urbanism by focusing on the practical work of European smart cities and community innovation partners, promoting everyday experiences rooted in the public interest such as the right to the city and the idea of sharing, thus reshaping the practice of a “citizen-centred” smart city discourse [8].

Based on smart cities as a global issue involved in local practice, the issue of how to develop rationalized standards and norms for smart cities and the dynamic competition between world cities and second and third tier ‘follower’ cities have become globally engaged issues. To some extent, the global character of smart cities and the transformative governance issues they represent point the way to future research and policy practice [9]. Through the construction of three-dimensional economic, social, and environmental indicators, 173 countries were examined in terms of their sustainable development [10], ultimately providing a system of indicators for sustainable development. Little is known about how the norms and perceptions of institutional arrangements influence and shape urban experiments. The institutional planning of smart cities in Amsterdam, Hamburg, and Ningbo was examined to explore how urban experiments are shaped based on local specificities, leading to the relationship between urban policy arrangements and concrete urban practices [11]. Technological rescue and urban crisis as a narrative premise of a smart city have become a socio-technical imaginary concept, breaking the narrative discursive status of the technology-dominated smart city imaginary and opening up space for a humanistic urban narrative [12]. Smart city research in Canada revolves around large cities and is very limited in targeting rural and remote development. How to establish communication between the development of lagging areas and big cities and how to synergize the smart development of rural remote areas are necessary to bridge the digital divide as well as for scale development [13]. The construction of public spaces in smart cities requires the construction of spatial infrastructures that combine virtual and real, organically combining the physical tangible space and virtual digital space of the city to further enhance the level of urban services and governance. Faced with the development needs of an intelligent and humanized city, the key to upgrading the construction of urban spatial infrastructure lies in the synergistic coupling of functional mechanisms, the use of digital intelligence technology to model and analyze urban spatial realities, and the shaping of a comprehensive governance system that is agile and responsive, with responsible subjects in place and the effective integration of resources [14]. The urban social structure and governance system should be reshaped intelligently, and the systematic scientific thinking paradigm should be used to guide the intelligent transformation of urban governance to achieve the goal of good urban governance. The logic of intelligent technology governance is integrated with urban governance to explore the role of systematic, holistic, self-organized, and collaborative behavior in urban innovation governance, to build an urban intelligent governance mechanism, and to adapt to the complexity requirements [15]. Under the background of new crown epidemic prevention, urban smart governance is based on the dual logic of collaborative governance and technological innovation. Taking the innovation of health code technology as an example, combined with the construction of digital governance ecosystems such as government governance and grassroots governance, governance innovation has emerged [16].

Although international research on smart cities started earlier, Chinese smart city research has just begun. In terms of research content, Chinese smart city research is more focused on technological innovation and infrastructure development, while international research is broader, covering technology, policy, and humanities; at the same time, international research is more concerned with the economic value derived from smart city construction, while Chinese research is more concerned with its public value. Many studies have addressed the conceptualization of the smart city itself, with one illuminating definition being that a smart city is a system integration of technological infrastructure that relies on advanced data processing with the goals of making city governance more efficient, citizens happier, businesses more prosperous, and the environment more sustainable [17]. In view of the current situation that China’s smart city research consists mostly of theoretical overviews, qualitative analyses or experience summaries, this paper uses bibliometrics as a tool to explore the overall research context, existing deficiencies, and future research trends to provide theoretical reference for the research and construction of smart cities at home and abroad.

## 3. Research Design and Integrity Analysis

**Figure 1:**
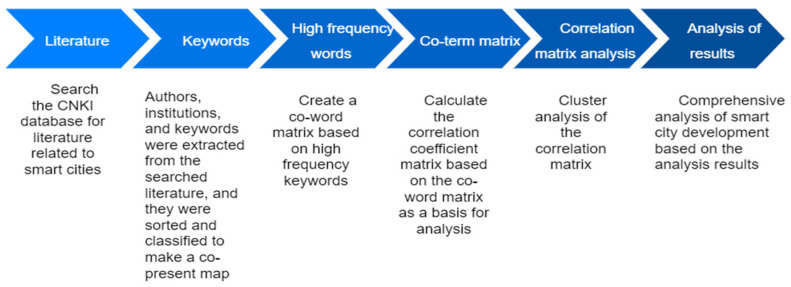
Research technology roadmap.

Bibliometrics is a cross-cutting science field that uses mathematical methods of mathematical statistics to analyze the literature in a field of interest. VOSviewer is commonly used in bibliometrics as visual mapping software to structure and visualize the knowledge domain of the literature by extracting data units and constructing a visual spectrum of the relationships between data. The SPSS statistical analysis process includes descriptive statistics, general linear models, regression analysis, log-linear models, cluster analysis, time series analysis, multiple responses, etc. COOC software is mainly used for literature database de-duplication, co-occurrence matrix, two-mode matrix, similarity matrix, etc. Bibliometric tools are commonly used in various disciplines of social and natural sciences to achieve a holistic grasp of trends and the current state of research in related disciplines through the processing of large amounts of data from relevant literature. This study is a bibliometric approach to data analysis of the literature related to "smart cities", and the research design is broadly divided into the following sections (Figure 1): (1) Retrieve smart city-related literature from the CNKI database; and on this basis (2) extract authors, keywords, time, journals, and other categories and make a co-occurrence map. (3) Establish a co-word matrix according to high-frequency keywords, (4) calculate the correlation coefficient matrix according to the co-word matrix as the basis of the analysis, and (5) carry out cluster analysis on the pre-relation matrix, (6) according to the cluster spectrum map to conduct a comprehensive analysis of smart city development.

### 3.1. Data Sources

The data used in this paper were obtained from the CNKI database. As of 31 March 2022, the title, key words, and abstract containing “smart city” were selected. The source categories of journals were searched, i.e., Peking University core, CSSCI journals, and CSCD journals, and a total of 2236 documents were retrieved. After the literature was processed (excluding studies that lacked keywords, meeting notices, and studies that lacked authors and years), 1976 documents were finally obtained as sample data.

### 3.2. Research Tools

Co-word analysis uses SPSS, COOC, VOSviewer, and other software to analyze the degree of association between words, such as keyword clustering, second mock examination co-occurrence analysis, hierarchical clustering, and so on, to outline the current situation of smart city research. Co-word analysis shows the relationship between the research objects through data and images, then summarizes the relevant literature, and discusses the research status and future development trend of smart cities.

### 3.3. Overall Research and Analysis

In this study, the year, author, journal, and high-frequency keywords were analyzed through bibliometric metrics. Specifically, (1) the annual publication of China’s smart city research was analyzed, (2) VOSviewer, COOC, and other software were used to analyze co-appearing authors and keywords and journals and keywords, and then the two-model co-occurrence relationship between related authors, journals, and keywords was excavated, and (3) multi-level clustering analysis and keyword clustering co-occurrence analysis were carried out for high-frequency keywords.

#### 3.3.1. Time Distribution Analysis

**Figure 2:**
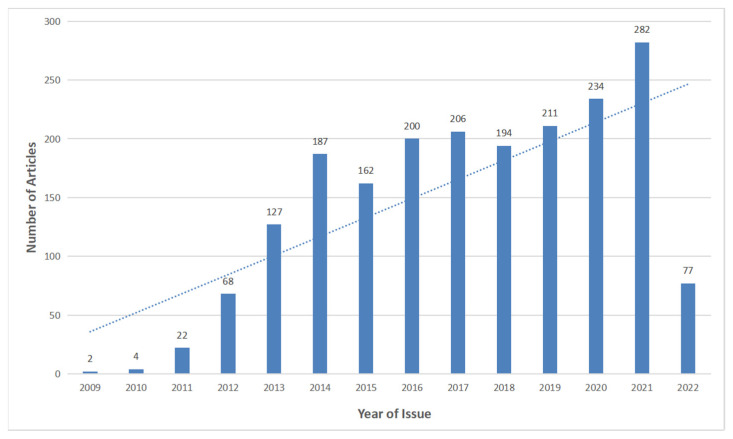
Time distribution chart.

According to the time distribution map of smart city research literature (Figure 2), China’s smart city research showed a steady increase and an upward trend year by year. The historical trend of the number of articles published was roughly divided into three stages: in the first stage (2009–2012), the number of articles published showed an exponential growth, reaching 68 in 2012. In the second stage (2013–2015), the number of documents fluctuated around 150, maintaining a stable trend. In the third stage (2016–2022), the number of documents basically fluctuated at a high level of 200 and reached a historical peak of 282 in 2021.

#### 3.3.2. Co-Occurrence Analysis of Authors and Keywords

**Figure 3:**
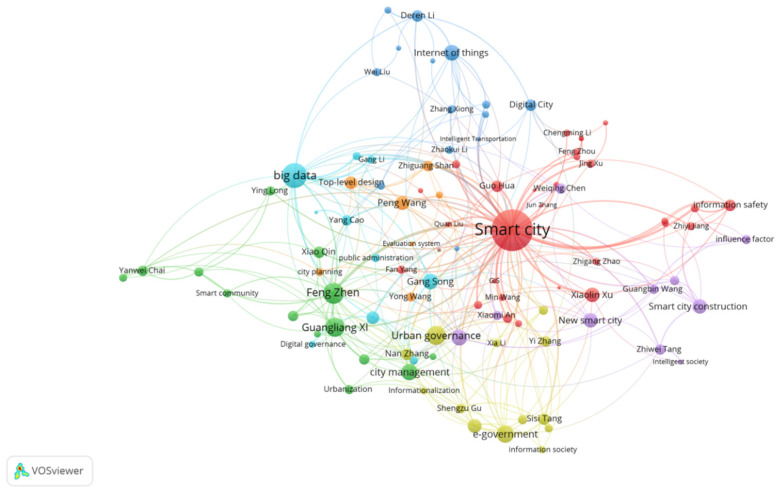
Co-occurrence atlas of authors and keywords.

There were 54 authors whose frequency was greater than or equal to five times, and 51 keywords whose frequency was greater than or equal to 10 times. The second mock examination of the co-occurrence of authors (54) and keywords (51) (Figure 3) represents the number of simultaneous occurrences. The larger the circle in the figure, the higher the frequency. This visually presents the areas highlighted by the author’s research. As shown in Figure 3, Deren Li, Wei Liu, and others published relevant research in the fields of the Internet of things, intelligent transportation, and digital cities. Guo Hua, Chengming Li, Feng Zhou, Jing Xu, Zhigang Zhao, et al. have published relevant articles on smart cities and information safety. Guangbin Wang and Zhiwei Tang have highlighted relevant research on smart city construction, new smart cities, and so on. Shengzu Gu, Sisi Tang, and others have focused on information society and e-government. Feng Zhen, Guangliang Xi, Xiao Qin, and Yangwei Chai have studied the smart community, urbanization, and so on.

#### 3.3.3. Analysis of the Co-Occurrence of Journals and Keywords

**Figure 4:**
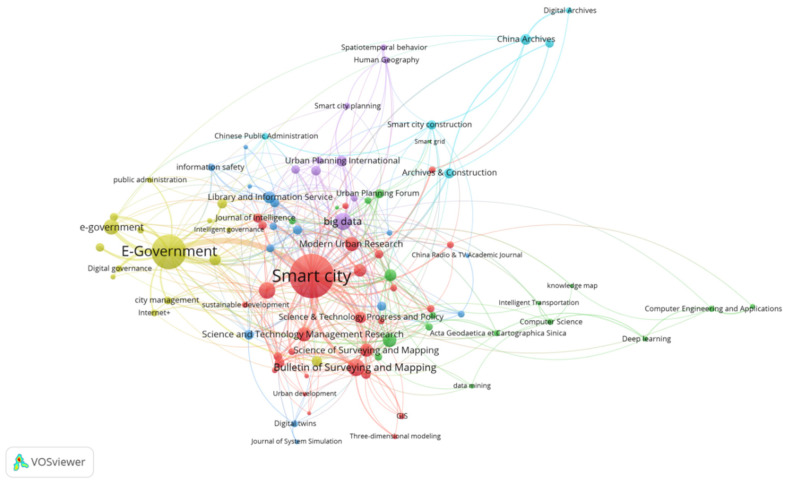
Co-occurrence map of journals and keywords.

The 41 journals have published 10 or more articles. In addition, the 51 keywords had a frequency of 10 or more times. Visual analysis of the second mock examination of the co-occurrence between journals (41) and keywords (51) was carried out, and the results are shown in Figure 4. “Computer Engineering and Applications”, “Computer Science”, and “Acta Geodaetica et Cartographica Sinica” focus on artificial intelligence, knowledge map, intelligent transportation, data mining, deep leaning, etc. Journals such as “Archives & Construction” and “China Archives” focus on key words such as digital archives, smart grid, smart city construction, etc. “Science and Technology Management Research”, “Modern Urban Research”, “Science of Surveying and Mapping”, and “Bulletin of Surveying and Mapping” focus on key words such as smart city, sustainable development, etc. “Urban Planning International”, “Human Geography”, and “Urban Planning Forum” focus on smart city planning, spatio-temporal behavior, big data, and other aspects. “E-government” focuses on intelligent governance, Internet+, etc.

#### 3.3.4. High-Frequency Keywords

The keyword fields of the 1976 documents were measured by COOC [18] software carrying out word frequency statistics, and 4977 keywords were extracted, including 51 keywords with a frequency ≥10. COOC software was used to generate 51 high-frequency keywords, and the co-occurrence matrix of 51 (Table 1) showed some high-frequency keywords due to layout constraints. In order to facilitate multi-scale analysis and hierarchical cluster analysis, the keyword co-occurrence matrix was transformed into a dissimilarity matrix by COOC software (Table 2).

#### 3.3.5. Cluster Analysis

**Figure 5:**
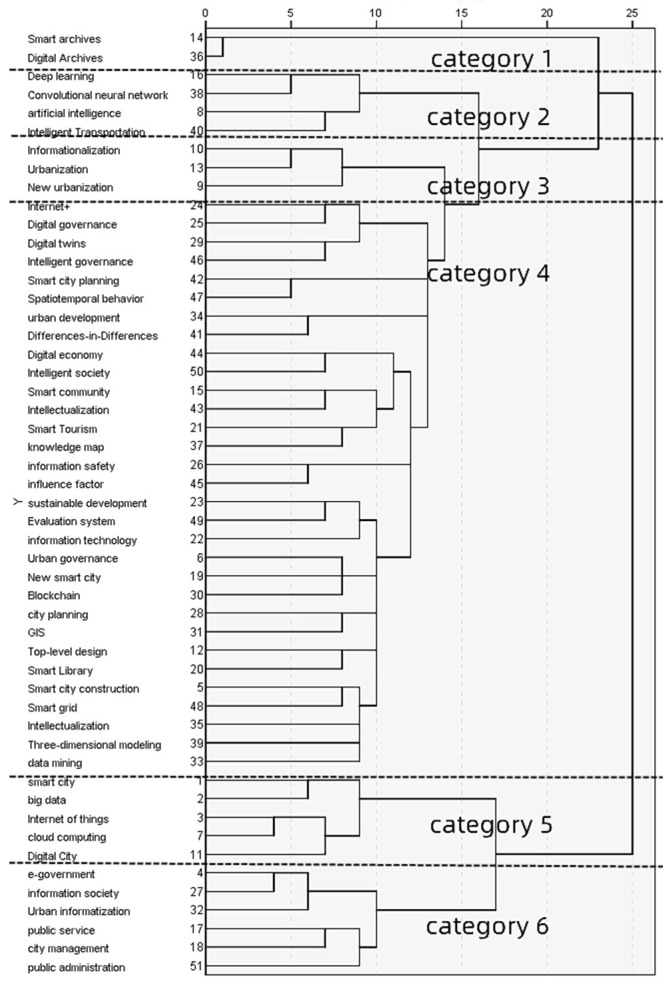
Hierarchical cluster analysis of high-frequency keywords.

The 51 keyword dissimilarity matrix (Table 2) in the CNKI database was imported into SPSS25 software, and the pedigree of the word connection is used to cluster the high-frequency keywords into six categories (Figure 5). Category 1 focused on archived research; category 2 focused on embedded research such as artificial intelligence technology; category 3 focused on urbanization; category 4 focused on smart city planning, construction and development, governance digitization, and other fields; category 5 focused on research related to smart cities and big data Internet of things; and category 6 focused on research related to e-government, urban management, and public services.

**Figure 6:**
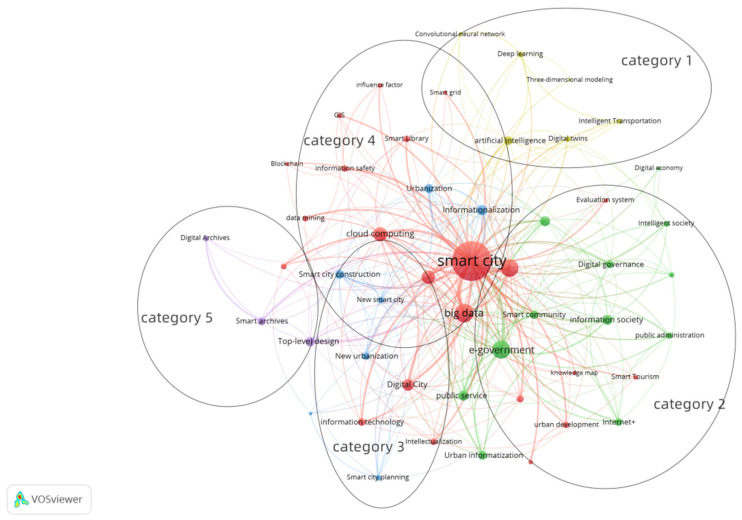
High-frequency keywords clustering co-occurrence mapping.

The co-occurrence matrix of 51 keywords from the CNKI database (Table 1) was imported into COOC software and converted to a net format while the co-occurrence profile was derived from VOSviewer software (Figure 6). The clustering of high-frequency keywords resulted in five categories: category 1 (yellow circled dots in the figure) included keywords such as digital twins, artificial intelligence, convolutional neural network, deep learning, three-dimensional modeling, intelligent transportation modeling, and intelligent transportation. In category 2 (green circled dots in the figure), the keywords were public service, information society, digital governance, city management, Internet+, urban informatization, public administration urban informatization, public administration, e-government, intelligent governance, digital economy, intelligent society, smart community, etc. The category 3 (blue circled dots in the diagram) keywords were new smart city, informationalization, spatiotemporal behavior, smart city planning, smart city construction, urbanization, new urbanization, etc. The category 4 (red circled dots in the figure) keywords included smart city, big data, cloud computing, Internet of things, data mining, urban development, digital city, blockchain, evaluation system blockchain, evaluation system, urban governance, sustainable development, intellectualization, city planning, information safety, smart grid, GIS, smart tourism, etc. The key words in category 5 (purple circled dots in the figure) were smart archives, top-level design, and digital archives.

Keyword clustering in Figure 6 was analyzed, which showed that category 4 (red keywords in the figure) intersected with categories 1, 2, and 3; this was clearly seen from the keyword clustering space in the figure. To a certain extent, this also confirms that the hierarchical clustering effect of high-frequency keywords in Figure 5 is not ideal, but this does not prevent us from introducing the relevant research topics in this study. We identified the research topic of classification 1 in the cluster diagram as intelligent technology supporting smart city research. The research topic of classification 2 was the integration of the social system of a smart city. The theme of category 3 was research in the fields of top-level strategic design and the planning and construction of smart cities. The research topics of classification 4 and 5 were the research of smart city construction development, evaluation, and specific practice.

## 4. Results and Discussion

According to Figure 6, the five categories are discussed according to four themes. The four themes are the research on smart cities supported by smart technologies, research on the integration of social systems in smart cities, research on the top-level strategic design, planning, and construction of smart cities, and research on smart city construction and development, evaluation, and specific practices.

### 4.1. Smart Technology to Support Smart City Research

The application of the latest technology in the Internet of Things era has accelerated the construction of urban infrastructure and has realized human–computer interaction and human–human interaction through network transmission, bringing convenience to human production and life. Unfortunately, however, the contradiction between user privacy and forensic investigation is inevitable. How to protect privacy and security based on forensic services requires corresponding solutions to provide a reasonable framework to ultimately ensure the security of data flow across domains [19]. Data drive the operation of smart city application systems, and the effective mining and development of data in smart city application systems are related to the high-quality development of smart cities. However, these data are usually sensitive and private, and the trade-off between data privacy protection and data availability has become a challenge; through privacy computing, federated learning, and other methods for data mining and the utilization of smart cities, data security is finally ensured under the premise of the availability and value of data [20]. The application development of the edge computing paradigm provides an efficient computing network system for the development of urban society, constructing an open platform for computing, storage, application capabilities, etc., providing a technical computing framework as well as a computing service system for the future development of urban wisdom. Heterogeneous intelligent drones can realize a differentiated framework for monitoring cities and oceans. The environment of smart cities and oceans is very different, and the monitoring of public transport related to smart cities can be realized through a tight flat-based framework, while at the same time a loose hierarchy-based framework is developed to realize the monitoring of oceans, which provides a technical idea for the smartization of marine cities [21]. The underlying technology to support smart cities requires multi-interface access and the signal processing of massive heterogeneous IoT devices based on artificial intelligence algorithm technology to achieve performance enhancement and improved processing power to realize the beautiful blueprint of smart cities [22]. This could strengthen the risk management and control of smart city network systems, using technical means to reduce risks or pass on risk losses through third-party network insurance companies [23].

There is an “innovation alienation trap” in the science and technology innovation cycle. The irrational perception of the science and technology innovation cycle, the purpose of innovation, and the relationship between the city and society has led to problems in the process of using science and technology for smart city construction. It is necessary to understand the characteristics of the science and technology innovation cycle and its value objectives, to accurately grasp the scale of innovation, to create a good innovation space, to stimulate innovation, and to achieve a sustainable model of smart city development [24]. The construction of Japan’s super-smart society in the context of innovation system theory is led by government policy, with universities cultivating relevant knowledge and talents, and the collaborative development of research, industry, and academia through the industrialization of enterprise technology, which provides corresponding guidance suggestions for the construction of smart cities in China [25]. There are many security risks in the construction and operation of smart cities, such as the lack of top-level network security design, long-term restriction of core technology products, insufficient investment in digital network security, and a lack of awareness of digital network security protection. The construction of a risk assessment model for smart cities will be beneficial in promoting urban risk prevention [26].

### 4.2. Research on the Integration of the Social System of a Smart City

The “true wisdom” of smart city construction in the new crown epidemic period needs to be people-centered, and it needs to turn “crisis” into “opportunity”, “individuality”, and “collectivity” integration and to create an intelligent governance model of multi-subject co-governance [27]. Major public health events affect the quality of urbanization development and promote the transformation of urban development from “population–spatial expansion” to “function–ecological connotation” [28]. It is beneficial to change the orientation of local government performance evaluation, build a multi-level urban governance system, deepen the construction of the social functions of urban agglomerations and smart cities, improve the level of high-quality urban development, and prevent and control public health incidents. Public emergencies test the level of smart city governance and help the transformation of urban governance from the aspects of big data prediction service capabilities of smart cities, urban emergency management coordination capabilities, smart city emergency material support capabilities, smart city refuge space planning and construction capabilities, and the management resilience of grassroots smart communities [29]. New infrastructure drives the construction of smart cities, from the iterative development of technology to urban space construction, changing the urban space network system and innovative practice application scenarios [30]. The construction of smart communities in the post-epidemic era has played a fundamental role in the urban circulation and people’s livelihood security system [31], and the precise prevention and control of the emergency management system for the prevention and control of the new crown epidemic is inseparable from the transformation of urban public management into the construction of smart cities, promoting the sharing of big data and strengthening information security construction [32].

Research on specific practice areas of smart city society: The concepts of “Industry 4.0”, “Society 5.0”, and “Connected Industry” have gradually become the consensus of modern social development, promoted the construction of the national strategic extension framework system of smart cities, taken suitability, technology, comprehensiveness, and systematization as the basic requirements for improving the development of smart cities, and explored strategic docking and cooperation such as science and technology strategies and industrial enterprises [33]. From the manufacturing transformation dimension, the design principles of Industry 4.0 include interoperability, virtualization, localization, real-time talent, service-oriented, and modular aspects [34]. The development of 5G technology provides a basic framework for the underlying framework for the development of smart cities such as the Internet of Things. Nanogenerators have become sustainable power sources and self-powered active sensors, which have contributed to smart city applications such as smart transportation and smart healthcare [35]. The complexity of smart city assessment indicators affects the decision making process for smart cities, and the development of a framework system for smart city assessment (named “SMART-C”) will help to promote smart cities [36]. The smart city environment monitoring system based on ZigBee wireless network forms a dynamic organization network through street lights, taxis, and other nodes to realize the infrastructure construction of a smart city with wireless network sensors, providing comfort and convenience for people’s lives [37]. Urban social governance is the nerve endings of urban governance, and it is indispensable to promote the modernization of urban governance. The urban resilience governance model is inseparable from the localization of the social development power mechanism, among which institutional resilience, economic resilience, and social resilience need to be further refined in the construction of urban space [38]. The disruptive technologies in the intelligent age have a transformative impact on urban space life, shaping urban science theories and the future development trend of smart cities. The development of smart cities promotes the modernization of urban governance systems and capabilities, realizes smart empowerment, and then resolves the contradiction analysis between technical governance and administrative governance, coordinates social relations in an orderly manner, and solves traditional urban problems [39].

### 4.3. Research in the Fields of Top-Level Strategic Design and Planning and Construction

Urban planning and design, construction, development, evaluation systems, and other related research: Smart city construction is inseparable from digital infrastructure, and competitive advantages should be ensured in the fields of top-level design and core technology research and development. Compared with latecomer countries, the US digital infrastructure does not have an advantage [40], and the lessons learned by the United States have reference value for the proportion of China’s new infrastructure structure, talent training, and urban–rural integration. From the macro strategic level, the US smart city construction strategy is based on four levels: top-level design, institutional mechanism, application deployment, and project practice, so as to provide a reference for China’s smart city construction [41]. Urban planning in the intelligent era needs to seek changes from passive adaptation to technology to active response, actively cater to the impact of intelligent technology on the development of disciplines, explore a new development system of urban planning, interpret the essence of smart city planning, clarify its positioning and development goals, sort out the four aspects, i.e., technical support, planning scheme, operation implementation, and system guarantee, explore the problems and contents of smart city planning at different spatial scales, and promote the intelligent transformation of urban planning [42]. The whole-process governance strategy consists of source control in advance, overall implementation during the event, and post-event assessment and evaluation, sorting out the functional architecture of the smart city system, monitoring system indicators, as well as platform simulation calculation, and data application in the smart city governance system, focusing on the digital support system in smart city planning, construction, operation, and maintenance management, realizing the data flow transfer and platform governance of smart cities, and providing a reference for smart cities and city brain operation and the maintenance management, assessment, and evaluation. For example, the problems existing in the construction of a smart city in Guangzhou are analyzed at five levels: strategic level, social activity level, economic activity, spatial structure, and technical support level, and corresponding reference suggestions are provided for planning and design, social management, people’s livelihood, industrial development, and the spatial structure layout [43].

Sustainable urbanization and informatization have become the trends of urban social development, and the use of informatization means to promote the sustainable development of urbanization and realize a low-carbon and sustainable urbanization ecological resilience system [44]. Urban geography research focuses on urban agglomerations, urbanization, smart cities, and other research fields, focusing on regional coordination, urban–rural integration, green development, etc. [45]. Urban development transformation is based on the original urbanization construction, should pay attention to infrastructure construction, urban functions and new technology integration, innovative development, green ecological construction, urban culture construction, and other aspects of upgrading and optimization, and finally create a livable, innovative, green, smart, and humanistic urban goal construction [46]. Urbanization development faces many urban problems, such as environmental pollution, imperfect infrastructure, and transportation, and the smart city construction process should respond to these “urban diseases”. The boundary of smart city construction is dominated by the value boundary of the city, and the value orientation of grasping the law of urban construction is the key to the development of a smart city. In the process of urbanization, information and communication technology (ICT) focuses on the individual spatial behavior of urban residents, among which social fairness, fairness and justice, and other issues need urgent attention [47]. The development stage, governance model, construction and management mode, service effect, and other aspects of the construction of new smart cities have changed, and policy suggestions for the development of new smart cities have been put forward and problems have been pointed out [48].

### 4.4. Research on Smart City Construction Development, Evaluation, and Specific Practice

We need to promote the construction of smart cities and explore the realization path of smart governance based on four dimensions: governance concept, model, system, and mode [49]. We need to carry out smart city construction pilots, improve governance systems and mechanisms, vigorously cultivate innovative talents, build digital platforms, shape a diversified and co-governance pattern, and jointly build a people-oriented sustainable smart city [50]. The digital transformation of cities has become the basic consensus of China’s smart city construction. Under the background of digital transformation, the governance of international metropolises follows the logic of scientific, intelligent, and refined governance, attaches importance to the overall planning of urban public data facilities, realizes the sharing of data resources, standardizes systems and rules, improves governance capabilities, and promotes the efficiency of metropolitan governance. Digital twin generation is a new aspect of smart cities and focuses on the field of digital city governance. A digital twin contextualizes traditional urban governance problems, intelligent methods, simplified processes, multiple subjects, and real-time interactions, but the dilemma brought about by digital twins to urban governance is not limited to technology, organization, value dilemma, etc., and it is necessary to explore the system optimization of digital twin urban governance from the aspects of top-level system design, organizational structure optimization, and people’s subject value anchoring [51]. The smart city theory based on digital twins is helpful to promote the solution of problems faced by urban development and the improvement of urban fine governance capabilities, realize the construction of urban data asset management systems, and implement digital business scenarios. Achieving refined governance in urban development in the new era is a key step in the urban governance system, which is an important link to promote the governance system regarding the details and depths, among which “technology empowerment” and “institutional guarantee” provide an integrated framework for urban fine governance, ensure the design of smart city systems and the construction of comprehensive administrative law enforcement systems, and promote urban fine governance [52].

We need to promote the construction of smart cities, the digital layout of global scenarios, and realize the construction of standardized systems for digital society and digital community infrastructure. We need to promote urban digitalization and refined governance, solve the problem of community governance application scenarios, and drive the development of science and technology industries related to urban construction [53]. Enterprises are the main body and driving force of urban economic development; enterprise intelligent operation, innovation and creation, precise services, etc., have become the characteristics of smart enterprises, and their development logic can be summarized as an “eight self-mechanism” (self-assessment, automatic perception, automatic prediction, self-correction, independent decision-making, independent evolution, self-improvement, automatic development), and the quality of intelligent enterprise operation and development ultimately creates value for users [54]. The theoretical framework of a library smart service uses the diversified services of information technology practice through the logical main line of “smart city—smart library service—user” and constitutes the theoretical framework of smart service from five aspects: macro analysis, specific element analysis, technical equipment, service innovation, and service landing [55]. The functional positioning of traditional comprehensive archives driven by smart city construction is reflected in precise services and accurate demand perception, integrating diversified data service functions, supporting urban cultural service functions, realizing intelligent services for archival information, and then constructing a relevant service network framework [56]. The high-quality development of smart file construction needs to be integrated into the construction of smart cities with the humanistic concept of benefiting the people [57]. The digital transformation of urban construction archives is conducive to the planning and construction of smart cities, reducing the corresponding approval links through intelligent innovative services, improving the legal administration system, promoting intelligent administration and intelligent services, and achieving the government affairs goal of one-network management [58].

## 5. Conclusions

In this paper, the selected literature data were limited to the CNKI database, and international databases were not comparatively studied, which makes the research data sample too localized and does not truly reflect the international value of China’s smart city research. In the next step of our research, we should strengthen the use of international databases to promote the international integration of China’s smart city research discourse system. Smart city research has attracted extensive attention from many disciplines, including urban science, management science, geographic science, urban sociology, economics, and other multidisciplinary fields. China’s smart city research focuses on the essential connotation, historical development, influential factors, logical framework, industrial application, and other fields of smart cities. In the future, the research on smart city construction should focus on the practical exploration of interdisciplinary system integration, urban digital simulation, dynamic mechanisms, and so on [59]. Through high-frequency keyword co-occurrence and cluster analysis, we visually measured the smart city research literature (a total of 1976 articles) from 2009 to 2022 (as of 31 March), reflecting the research status of related topics of a smart city in China. The construction and development of a smart city can be summarized by the following aspects:

First, the research theme of China’s smart city shows a tendency of scattered fragmentation. To a certain extent, there is no unified framework for domestic smart city research, which shows that smart city theory is in the initial stage of exploration, and its connotation essence has not reached a consensus, and the smart city framework under different theoretical frameworks is also different. Among them, the construction of smart cities from the perspective of system theory generates a holistic intelligent system through the coupling of three major systems: physical city, social city, and digital city, so as to realize digital, sustainable development and creative city construction [60]. From the perspective of urban development, a smart city is an organic and unified development model such as the economy, society, and ecology, and its development path realizes the development and construction of a smart city through the interaction of urban citizen knowledge, environmental intelligence, and technological intelligence [61]. From the perspective of the urban operation mechanism, a smart city is a new concept and new model that uses convergent technologies such as big data, cloud computing, and artificial intelligence to promote intelligent development such as urban planning, construction, and services.

Second, the law of smart city construction and development needs to be further explored. For example, urban governance, especially the governance of megacities, needs to explore a social governance path that conforms to the characteristics of the development laws of megacities, pay attention to the construction of a digital and intelligent urban governance system, improve the level of urban services, and make cities more agile, intelligent, ecological, and livable. At present, population aging has become a development trend in China. How to use big data to drive the precise and intelligent development of the traditional pension model has become a real problem. The big data platform can accurately meet the needs of the elderly and guarantee that the flow of data, capital, and service is coordinated and unified, the allocation of resources is optimized, and the healthy and orderly development of the aging society is realized [62].

Third, the philosophical humanism and ecological ethics of smart cities need to be systematized. The risks encountered in urban governance in the digital era need to be considered from the overall nature of urban society, and issues such as technical risks and the nature of human practice should be examined from the perspective of urban ethics to provide reasonable space for the innovation of the co-construction system of smart city construction [63]. The coating theory of Chinese scholar Professor Chen Zhong provides a corresponding theoretical framework for humanistic city research, and it is of great benefit to avoid alienation and coating formalism [64]. Urban smart life is the combination of a smart city and an ecological city, and it is an inevitable trend of future urban development. There is a need for the construction of smart cities to follow the perspective of the ecological environment. In the process of the digital transformation of a smart city, for example, the low-carbon travel of shared bicycles contributes to ecological environment governance. Through the analysis of relevant air quality testing data, the practical value of a smart and green city is presented [65].

Fourth, the organic life form of a smart city needs to be nourished by theories related to global cities, innovative cities, and urban innovation. The world city, a product of globalization, is undergoing a transformation into a digital, intelligent organic city life form, a process that will inevitably bring about structural changes in society, which will in turn affect the industrial system of the urban space and issues such as citizen participation. The organic and innovative smart city is a system in which innovation agents, mechanisms, and environments interact with each other, with intrinsic dynamic mechanisms driving the sustainable economic, social, and cultural development of the city.

Finally, smart cities may reshape the future of urban development, and the technocratic-induced critique of smart cities will become a new model for the vision of neoliberal urban governance. With the rise of the critical discourse on smart cities, the framework of the smart city vision needs to be redefined and combined with a case study of the smart city in Cologne, Germany, to demonstrate social innovation and the future changes of sustainable cities that are “genuinely” smart [66]. Smart city ecosystem is a multi-level complex structure; how to govern a smart city not only requires digital intelligent technology system as support but also needs to pays attention to the problems and challenges faced in daily life [67]. The discourse system of technology-driven smart cities has created the emergence of technological determinism, so the critical discourse of humanistic dimensions in specific spaces is conducive to technological innovation in urban development and the creation of the humanistic attributes of urban life [68].

## Figures and Tables

**Table 1:** High-frequency keyword co-occurrence matrix (part).

Key Word	Smart City	Big Data	Internet of Things	E-Government	Smart City Construction
smart city	983	68	63	55	12
big data	68	123	13	12	0
Internet of things	63	13	92	3	1
e-government	55	12	3	63	1
smart city construction	12	0	1	1	60

**Table 2:** High-frequency keyword dissimilarity matrix (part).

Key Word	Smart City	Big Data	Internet of Things	E-Government	Smart City Construction
smart city	−2.220446049250	0.804440194	0.790506826	0.778988101	0.950588406
big data	0.804440194	1.11022302462516	0.877792775	0.863680365	1
Internet of things	0.790506826	0.877792775	−2.22044604925031	0.96059448	0.986540452
e-government	0.778988101	0.863680365	0.96059448	1.11022302462516	0.983734999
smart city construction	0.950588406	1	0.986540452	0.983734999	1.11022302462516

## Data Availability

The raw data supporting the conclusions of this article will be made available by the authors without undue reservation.

## References

[1] Zhang X.J., Zhang Z.G. (2017). Construction and Development Countermeasures of smart city under the background of China’s urban society. Sci. Technol. Manag. Res..

[2] Gao X. (2017). Key points, difficulties and strategy selection of China’s smart city construction from the perspective of comparative analysis. Sci. Technol. Manag. Res..

[3] Johnson P.A., Acedo A., Robinson P.J. (2020). Canadian smart cities: Are we wiring new citizen-local government interactions?. Can. Geogr.-Geogr. Can..

[4] Esposito C., Castiglione A., Frattini F., Cinque M., Yang Y., Choo K.K.R. (2019). On data sovereignty in cloud-based computation offloading for smart cities applications. IEEE Internet Things J..

[5] Correia D., Teixeira L., Marques J.L. (2021). Reviewing the state-of-the-art of smart cities in portugal: Evidence based on content analysis of a portuguese magazine. Publications.

[6] Yigitcanlar T., Kankanamge N., Vella K. (2021). How are smart city concepts and technologies perceived and utilized? A systematic geo-twitter analysis of smart cities in australia. J. Urban Technol..

[7] Xie J., Tang H., Huang T., Yu F.R., Xie R., Liu J., Liu Y. (2019). A survey of blockchain technology applied to smart cities: Research issues and challenges. IEEE Commun. Surv. Tutor..

[8] Cardullo P., Kitchin R. (2019). Smart urbanism and smart citizenship: The neoliberal logic of ‘citizen-focused’ smart cities in europe. Environ. Plan. C-Politics Space.

[9] Joss S., Sengers F., Schraven D., Caprotti F., Dayot Y. (2019). The smart city as global discourse: Storylines and critical junctures across 27 cities. J. Urban Technol..

[10] Kaklauskas A., Kaklauskiene L. (2022). Analysis of the impact of success on three dimensions of sustainability in 173 countries. Sci. Rep..

[11] Raven R., Sengers F., Spaeth P., Xie L., Cheshmehzangi A., De Jong M. (2019). Urban experimentation and institutional arrangements. Eur. Plan. Stud..

[12] Sadowski J., Bendor R. (2019). Selling smartness: Corporate narratives and the smart city as a sociotechnical imaginary. Sci. Technol. Hum. Values.

[13] Spicer Z., Goodman N., Olmstead N. (2021). The frontier of digital opportunity: Smart city implementation in small, rural and remote communities in canada. Urban Stud..

[14] Sun X., Shan X.Z. (2021). Spatial Infrastructure Construction for Smart Cities: From Functional Synergy to Digital Synergy. E-Government.

[15] Zhang N., Yang J.Z. (2022). Research on Smart City Governance Innovation Based on Systems Thinking. J. Syst. Sci..

[16] Shi C., Ma L. (2020). Collaborative Governance, Technological Innovation and Smart Epidemic Prevention: A Case Study Based on “Health Code”. Party Gov. Res..

[17] Yin C., Xiong Z., Chen H., Wang J., Cooper D., David B. (2015). A literature survey on smart cities. Sci. China Inf. Sci..

[18] Academic Dots, Bibliometrics COOC a New Software for Bibliometrics and Knowledge Mapping [EB/OL]. (2020-01-12). https://mp.weixin.qq.com/s/8RoKPLN6b1M5_jCk1J8UVg.

[19] Stoyanova M., Nikoloudakis Y., Panagiotakis S., Pallis E., Markakis E.K. (2020). A survey on the internet of things (iot) forensics: Challenges, approaches, and open issues. IEEE Commun. Surv. Tutor..

[20] Qi L., Hu C., Zhang X., Khosravi M.R., Sharma S., Pang S., Wang T. (2021). Privacy-aware data fusion and prediction with spatial-temporal context for smart city industrial environment. IEEE Trans. Ind. Inform..

[21] Kim H., Mokdad L., Ben-Othman J. (2018). Designing uav surveillance frameworks for smart city and extensive ocean with differential perspectives. IEEE Commun. Mag..

[22] Peng W., Gao W., Liu J. (2019). Ai-enabled massive devices multiple access for smart city. IEEE Internet Things J..

[23] Sharma K., Mukhopadhyay A. (2022). Sarima-based cyber-risk assessment and mitigation model for a smart city’s traffic management systems (scram). J. Organ. Comput. Electron. Commer..

[24] Wu J. (2021). The Science and Technology Innovation Cycle of Smart Cities: Implications, Problems and Approaches. Explor. Controv..

[25] Guo Y.H., Tang Z.W., Zhao D., Wang Y. (2020). The enlightenment of Japan’s super-intelligent society under the national innovation system to the construction of China’s intelligent society. Sci. Technol. Manag. Res..

[26] Chen Y.H., Yang S.L., Li Y.G., Chen F.Q. (2020). Construction of smart city security risk assessment model and countermeasures. E-Government.

[27] Zhang J. (2020). How can smart city construction be “true wisdom”. People’s Forum.

[28] Zhao J. (2021). Major Public Health Events and the Quality of China’s Urbanization: Theoretical Framework, Evolution and Path Selection. Macro Qual. Res..

[29] Wang B., Zhang W., Zhang J.Q. (2021). Smart city construction and urban governance transformation under public emergencies. Sci. Technol. Rev..

[30] Wu Z.Q., He R., Xu H.W., Feng T.X., Zhang S.H., Yang T. (2021). On the iterative law of new infrastructure construction. Urban Plan..

[31] Wang C.R., Wang C.J. (2020). “In the post-new crown era”, Thinking on Accelerating the Construction of Smart Community Business Service System. J. Bus. Econ..

[32] Zhao Z.X., Hu B.B. (2021). Ideas and countermeasures for the digital transformation of emergency management system. Sci. Technol. Manag. Res..

[33] Zhang Y.X., Li G.C. (2021). A Review of Japan’s Industrial Science and Technology Strategic System in the New Era. Mod. Jpn. Econ..

[34] Oztemel E., Gursev S. (2020). Literature review of industry 4.0 and related technologies. J. Intell. Manuf..

[35] Yigitcanlar T., Kamruzzaman M. (2019). Smart cities and mobility: Does the smartness of australian cities lead to sustainable commuting patterns?. J. Urban Technol..

[36] Castanho M.S., Ferreira F.A.F., Carayannis E.G., Ferreira J.J.M. (2021). Smart-c: Developing a “smart city” assessment system using cognitive mapping and the choquet integral. IEEE Trans. Eng. Manag..

[37] Lv Z., Hu B., Lv H. (2020). Infrastructure monitoring and operation for smart cities based on iot system. IEEE Trans. Ind. Inform..

[38] Chen Y., Ge Y., Chen R.S., Chen W.J., Ye Z.C., Liu H. (2020). New progress in the research of foreign urban resilience under the background of climate change: Bibliometric analysis based on Citespace. J. Catastrophology.

[39] Li Q., Liu H.J. (2020). Smart cities and modernization of urban governance: From conflict to empowerment. Adm. Reform.

[40] Chen X.D., Li S. (2021). Status and challenges of digital infrastructure in the United States. Mod. Int. Relat..

[41] Zhu C.K., Wang Y.B. (2021). The development strategy and inspiration of smart city construction in the United States. Local Gov..

[42] Yan F., Kong Y. (2021). A framework for smart city planning with “people-technology-space”. J. Urban Plan..

[43] Zhang Z.G., Zhang S.J. (2015). The current situation, problems and countermeasures of smart city construction in Guangzhou. Sci. Technol. Manag. Res..

[44] Liu M.X., Ding Y. (2015). Research on the coordinated development of sustainable urbanization and information technology. Sci. Technol. Manag. Res..

[45] Yan F., Xu J.T., Xi G.L. (2021). Progress and prospects of urban geography research at home and abroad in the last decade. Econ. Geogr..

[46] Liu Z.X. (2021). Research on transforming the urban development mode to promote new urban construction. Econ. J..

[47] Feng J., Shen X. (2021). A review of information and communication technology (ICT) and urban geography. Hum. Geogr..

[48] Tang S., Zhang Y.Q., Shan G.Z., Wang W., Zhang Y.Q. (2020). Current status, situation and policy recommendations for the development of new smart cities in China. E-Government.

[49] Zhang H.M., Zhang H.Y. (2021). Exploring the path and strategy of urban wisdom governance. J. Hainan Univ. (Humanit. Soc. Sci. Ed.).

[50] Feng M.L. (2019). Human-oriented smart city planning and management based on time-space behavior. Open House Int..

[51] Xiang Y.Q., Xie X.S. (2021). Digital twin city governance: Changes, dilemmas and responses. E-Government.

[52] Li X.S. (2020). Reconstructing the logic of fine-grained urban governance in the new era: A “technology-enabled” perspective. Urban Dev. Res..

[53] Zhang R.B., Zhu X., Li X.Y. (2021). Smart city construction to promote innovation in community governance practices. J. Beijing Union Univ. (Humanit. Soc. Sci. Ed.).

[54] Zhang J.C. (2021). Smart enterprise: Background, characteristics, logic and concept. Enterp. Econ..

[55] Liao J.Q. (2020). A theoretical framework for core elements of library smart services. Library.

[56] Wei L.L. (2020). Public services of comprehensive archives in the context of smart cities: Functional positioning and participation mechanism. Beijing Arch..

[57] Lin Y.M., Yu Y.Y. (2020). Exploration and practice of building a wisdom archive in Weihai City. China Arch..

[58] Che T.T. (2020). The digital transformation of China’s urban archives under the layout of “digital China”. Arch. Constr..

[59] Yao C., Zhen F., Xi G.L. (2021). Progress and prospects of smart city research in China. Hum. Geogr..

[60] Xia H.X., Wang Z.T. (2017). Some thoughts on smart cities from a system perspective. China Soft Sci..

[61] Li C.J. (2015). Research on the connotation, characteristics and development paths of smart city—Example of the construction of smart city in Beijing. Mod. Urban Res..

[62] Jia Y., Lan Z.Y., Liu R.Z. (2020). Precise Aging: A New Model of Aging Driven by Big Data. J. Public Adm..

[63] Li F.F., Dong H. (2020). Ethical demands of urban governance in the era of big data. Urban Dev. Res..

[64] Chen Z. (2022). Coated urban renewal: How to generate and how to overcome?—Based on the perspective of humanistic urbanism and urban philosophy. Gansu Soc. Sci..

[65] Xiao Q.B., Chen L., Pei D. (2021). Sharing economy and environmental governance in smart cities: An example of low-carbon travel with shared bicycles. China Soft Sci..

[66] Leitheiser S., Follman N. (2020). The social innovation-(re)politicisation nexus: Unlocking the political in actually existing smart city campaigns? the case of smartcity cologne, germany. Urban Stud..

[67] Caputo F., Walletzky L., Stepanek P. (2019). Towards a systems thinking based view for the governance of a smart city’s ecosystem a bridge to link smart technologies and big data. Kybernetes.

[68] Odendaal N. (2021). Everyday urbanisms and the importance of place: Exploring the elements of the emancipatory smart city. Urban Stud..

